# CALENDAR

**DOI:** 10.1054/bjoc.1999.1191

**Published:** 2000-04-03


					2324 June 2000
II Melanoma Meeting for Specialists in dermatology,
surgery and medical oncology
Milan, Italy
Further information from:
Scientific Secretariat, Dr Alessandro Testori, European Institute
of Oncology, via Ripamonti 435-20141 Milan, Italy. Tel : + 39 02
57489 493; Fax : + 39 02 57489878; E-mail : atestori@ieo.it
or Organizing Secretariat, M.A.F. Servizi Srl, via G.B. Vico 7,
10128 Torino, Italy. Tel : + 39 011/505900; Fax : + 39 011/505
976; E-mail: melanoma2000@mafservizi.it
1013 July 2000
29th British Congress of Obstetrics and Gynaecology
International Convention Centre
Birmingham, UK
Further information:
BCOG Secretariat, Congress House, 65 West Drive, Cheam,
Sutton, Surrey SM2 7NB, UK. Tel: + 44 (0) 20 8661 0877;
Fax: + 44 (0) 20 8661 9036; E-mail: info@conforg.com
27 September  1 October 2000
Pigment Cell research - Perspectives for the Third
Millenium
University of Ulm/Donau, Germany
Further information from:
Department of Dermatology, University of Ulm, ESPCR 2000,
89070 Ulm/Donau, Germany. Tel: + 49 (0) 731 50 22251;
Fax: + 49 (0) 731 50 23772; E-mail: ralf.peter@medizin.uni-
ulm.dei; Website: http://www.uni-ulm.de/klinik/dermatologie
2830 September 2000
3rd Congress of the German Society of Palliative
Medicine
Gottingen, Germany
Further information from:
Kongress - Sekretariat, Frau G. Albrecht, Zentrum
Anaesthesiologie, Rettungs - und Intensivmedizin oler Georg -
August - Universitat Gottingen, Robert - Koch - Strasse 40,
D - 37075 Gottingen, Germany. Tel : + 49 551 39 88 26;
Fax : + 49 551 39 86 76; E-mail : gahlbre @gwdg.de
Announcement
The first official version of the Familial Cancer Database (FaCD),
written by the Dutch clinical geneticist Rolf Sijmons (University
of Groningen) and pathologist Gerard Burger (SAZINON
Foundation), has now been released in support of the UICC
Familial Cancer and Prevention Project
(http://www.uicc.ch/familial). FaCD is a stand-alone computer
program which may help physicians in diagnosing hereditary
cancer. It tries to match the phenotype of a cancer patient/family
with the profiles of the more than 300 cancer associated familial
and hereditary disorders stored in its database. FaCD is aimed at
health care professionals with at least a basic knowledge of
clinical cancer genetics. The software can be downloaded after
free registration at http://facd.uicc.org . The authors can be
contacted at facd@medgen.azg.nl
CALENDAR
1618
British Journal of Cancer (2000) 82(9), 1618
(c) 2000 Cancer Research Campaign
Article no. bjoc.1999.1191, available online at http://www.idealibrary.com on

				

